# Kinect-Based Assessment of Lower Limbs during Gait in Post-Stroke Hemiplegic Patients: A Narrative Review

**DOI:** 10.3390/s22134910

**Published:** 2022-06-29

**Authors:** Serena Cerfoglio, Claudia Ferraris, Luca Vismara, Gianluca Amprimo, Lorenzo Priano, Giuseppe Pettiti, Manuela Galli, Alessandro Mauro, Veronica Cimolin

**Affiliations:** 1 Department of Electronics, Information and Bioengineering, Politecnico di Milano, Piazza Leonardo da Vinci 32, 20133 Milano, Italy; serena.cerfoglio@polimi.it (S.C.); manuela.galli@polimi.it (M.G.); 2 Istituto Auxologico Italiano, IRCCS, Department of Neurology and Neurorehabilitation, S. Giuseppe Hospital, Strada Luigi Cadorna 90, 28824 Piancavallo, Italy; lucavisma@hotmail.com (L.V.); lorenzo.priano@unito.it (L.P.); alessandro.mauro@unito.it (A.M.); 3 Institute of Electronics, Computer and Telecommunication Engineering, National Research Council, Corso Duca degli Abruzzi 24, 10129 Torino, Italy; claudia.ferraris@ieiit.cnr.it (C.F.); gianluca.amprimo@ieiit.cnr.it (G.A.); giuseppe.pettiti@ieiit.cnr.it (G.P.); 4 Department of Neurosciences, University of Turin, Via Cherasco 15, 10100 Torino, Italy; 5 Department of Control and Computer Engineering, Politecnico di Torino, Corso Duca degli Abruzzi 24, 10129 Torino, Italy

**Keywords:** RGB-D sensors, gait analysis, stroke, hemiplegia, markerless motion analysis

## Abstract

The aim of this review was to present an overview of the state of the art in the use of the Microsoft Kinect camera to assess gait in post-stroke individuals through an analysis of the available literature. In recent years, several studies have explored the potentiality, accuracy, and effectiveness of this 3D optical sensor as an easy-to-use and non-invasive clinical measurement tool for the assessment of gait parameters in several pathologies. Focusing on stroke individuals, some of the available studies aimed to directly assess and characterize their gait patterns. In contrast, other studies focused on the validation of Kinect-based measurements with respect to a gold-standard reference (i.e., optoelectronic systems). However, the nonhomogeneous characteristics of the participants, of the measures, of the methodologies, and of the purposes of the studies make it difficult to adequately compare the results. This leads to uncertainties about the strengths and weaknesses of this technology in this pathological state. The final purpose of this narrative review was to describe and summarize the main features of the available works on gait in the post-stroke population, highlighting similarities and differences in the methodological approach and primary findings, thus facilitating comparisons of the studies as much as possible.

## 1. Introduction

Stroke and cerebrovascular diseases are leading causes of both death and long-term disabilities worldwide [1,2], and hemiplegia is the most common impairment in the survivors [3,4]. Stroke results in a wide range of neurological deficits [5], and it severely affects motor skills, causing muscular weakness or partial hemi-paralysis that compromises the full arm function [6] and the mobility of the lower limbs [5,7].

With respect to lower limbs, hemiplegic gait is the most common manifestation of stroke: it is characterized by inter-limb asymmetry in walking or dragging gait due to the unilateral weakness of the affected side, together with abnormal torso tilting rotation [5,8,9,10,11,12,13,14]. The decline in functional abilities and the impaired motor performance greatly affect the post-stroke patient’s quality of life [9,15,16,17]. In order to limit the consequent impact on people’s daily life, a timely assessment is crucial for proper monitoring of the improvement or worsening of motor skills, and to set up effective rehabilitation protocols fitting each individual’s condition [18,19,20]. In this context, specific rehabilitation protocols that assess the functionality of the trunk/pelvis and the joints of the lower limbs are desirable.

The assessment of gait-related impairments in hemiplegic post-stroke patients is commonly performed using standardized clinical scales (e.g., the Fugl-Meyer Assessment score) [21] and specific walking tests. Among them, there are the 10-meter walk test (10MWT) [22], the 6-minute walk test [23], and the Timed Up and Go (TUG) test [24], which are often performed as part of the same examination session to gain a complete description of the patient’s walking behavior [25]. In the clinical field, optoelectronic 3D motion capture systems and force plates are the broadly acknowledged gold standard tools to assess gait patterns in a laboratory setting due to their consistency and measurement accuracy [5,9,26,27]. Such systems provide spatiotemporal parameters, kinematics and kinetics that quantitatively describe the main features of the gait cycle, thus allowing for the functional performance assessment of patients and the identification of atypical patterns [5,28]. However, optoelectronic systems cannot be extensively used due to their high cost, complexity and often-troublesome equipment wearing requirements [29,30], together with the dependency on dedicated laboratory settings and specifically trained operators [26,31,32] and the need to wear limited clothing, which represents a great limitation for the application in many types of patients (for example, in patients with eating disorders or neurological patients with great functional problems in dressing).

Over the last decade, different technologies and methods [33,34,35,36,37,38,39,40,41,42] have been proposed as an alternative to the optoelectronic systems for the analysis of body movements and functions as a medical diagnostic tool as well as in sports assessment. Among them, low-cost optical body-tracking sensors, such as the Microsoft Kinect, have proved to be particularly promising in order to assess both healthy and pathological gait, posture, postural instability, and balance in a non-invasive way [5,9,43,44,45,46,47]. Originally featured for entertainment together with the Xbox^TM^ video game consoles, Microsoft Kinect^®^ (Microsoft Corporation, Redmond, WA, USA) has become a ground-breaking vision-based motion capture system, based on its color and depth sensors, finding application in new contexts including medical–clinical and rehabilitation settings [15,48]. Acknowledged as a non-intrusive tracking device [49], it requires neither any subject preparation nor attachment of markers to the patient’s body, nor a dedicated handheld controller [15]. In fact, its inherent technology is able to detect and capture the movements of the body in real-time by estimating the positions of the main joints through the anatomical landmarks of a skeletal model in the 3D space [50]. As it does not require any additional equipment, people are free to move with their natural patterns as they perform various tasks inside the device’s field of view, and their movements can be reproduced in real-time on the computer screen, for example, to obtain visual feedback [15]. The device is small, portable, and does not require a complex laboratory setup [26], enabling its use even as part of virtual home tele-monitoring and tele-rehabilitation systems [51,52,53,54,55,56] that may allow the patients to practice exercises in a private environment [3]. In addition, it allows for training in specific motor and non-motor tasks with an amusing game-based approach that may increase the motivation and engagement of the patients [15,57,58,59,60,61,62,63], in particular those in outpatient settings. This could permit an adequate and prolonged clinical monitoring in real-life contexts, thus replacing dedicated hospitalizations [64].

In recent years, several studies have investigated the accuracy and effectiveness of Microsoft Kinect for the assessment of posture, gesture, lower limbs and gait performance in several pathological states, such as stroke, Parkinson’s disease [26,65,66,67,68,69,70] and other pathologies [71,72,73,74]. Different studies have reported its reliability for the assessment of spatiotemporal gait parameters (e.g., step length and gait speed) and kinematic variables (e.g., trunk angle) in healthy individuals, with results comparable to those of laboratory-grade systems [25,75,76,77,78,79,80] using both the first and second model of the device. The last version of the device, the Azure Kinect DK, was released in 2019 and, thanks to its new body tracking algorithm based on deep learning and convolutional neural networks [81], shows improved features in terms of depth accuracy and the number of joints tracked compared to the previous generations. Pilot studies on gait analysis [81,82] highlight higher accuracy of the device in the estimation of spatial gait parameters and kinematics compared to the previous models. Recent studies found that Azure Kinect had good agreement with a traditional motion capture system setup, indicating that the sensor could provide clinically relevant measurement of spatiotemporal parameters during gait [83], postural control [84], and sit-to-stand movement strategies, allowing for improved precision in clinical decision-making across multiple clinical populations [85]. The results showed high levels of agreement in evaluating spatiotemporal and kinematic variables during walking, sit-to-stand, and functional balance tasks, indicating that this technology is capable of accurate, and clinically relevant, assessment of motion data while performing these tasks. However, to date, no studies have explored its applicability in the characterization of a pathological condition.

Kinect sensor has been extensively used in Parkinson’s disease gait analysis [65,86] and postural control tests [66], such as the single-leg eyes-closed standing balance [87], claiming concurrent validity with the gold standard systems [88] and showing its ability to accurately measure some temporal and clinically relevant spatial features [49]. This non-invasive optical sensor increased the odds of virtual reality in the rehabilitation. The use of exergames is an innovative viable strategy for rehabilitation purposes, because it is not only recreational, but it also allows one to stimulate cognitive and motor functions and to promote physical activities through a more engaged game interaction [89,90,91,92]. However, at present, few studies have used this technology in specific neurological treatments, such as in patients with stroke. The use of Kinect for the rehabilitation of post-stroke patients is in fact a recent topic. The first controlled and randomized studies were published in 2013 [93,94]. Moreover, the studies selected here represented small samples, and the majority comprised less than 30 volunteers. The greater use of Kinect with more significant results in the treatment of stroke patients was in the recovery of motor function and postural balance [95]. Nevertheless, conclusive findings on these and other variables were not yet possible, which increased the necessity for caution with this device in rehabilitation. With respect to the post-stroke population, however, just a few studies have focused on characterizing pathological gait patterns, but the lack of homogeneity among the characteristics of the cohorts, the selected pool of gait parameters, and the methodologies and objectives of the research make it difficult to directly compare their results.

This has led to uncertainties about the strengths and weaknesses of this technology to analyze and quantify gait patterns and walking strategies in post-stroke patients [18]. The aim of this narrative review was to present an overview on the state of the art regarding the use of the different Microsoft Kinect camera models to assess gait in post-stroke individuals through an analysis of the available literature. Both studies using this sensing technology to directly evaluate and characterize the gait patterns and studies aimed at its validation against gold standard references (i.e., optoelectronic systems) were considered and included.

## 2. Materials

An extensive search of the literature was performed in February 2022, with a focus on studies published over the past 12 years (2010–2022). The search was performed on Web of Science, PubMed, Scopus, Mendeley, and Google Scholar via customized queries using keywords and Boolean operators in the form “(Kinect OR Xbox) AND (Hemiplegia OR post-stroke) AND (Gait Analysis OR lower limbs)”. The document type was set to “Article” and the selection was limited to full articles written in English. Additionally, the bibliographies of the selected papers were manually checked in order to find other suitable studies. It was decided to include studies assessing only adults (>18 years) with hemiplegia, evaluating the functional limitation or the effects of rehabilitation with Microsoft Kinect. Studies focused on non-adult participants or in which the Kinect technology was not the main tool used to assess the participants were not included.

## 3. Results

**Figure 1:**
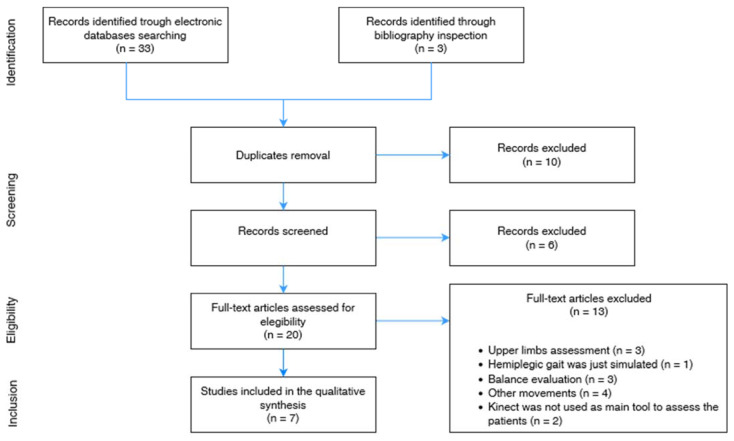
Process of study selection.

A total of 33 articles were retrieved from the above-mentioned electronic databases. Three items were added by visual inspection of refence lists of the selected studies. After removing 10 duplicates, title and abstract screening led to the exclusion of six papers. Out of the remaining 20 articles, 13 failed to meet the inclusion criteria. The selection process is shown in Figure 1.

Table 1 presents a summary of the papers on the use of Microsoft Kinect to evaluate the gait behavior of hemiplegic patients, together with the demographic characteristics of the evaluated cohort of patients, details regarding the experimental setup (analyzed gait parameters) and the purpose of the study, distinguishing between gait characterization, system validation or both.

The study carried out by Latorre et al. [18] included also non-adult participants (aged between 10 and 17 years), but as the authors worked with a large cohort of patients and the number of non-adult participants was small compared to the total number of participants, it was decided to include this study in the review.

The selected studies were analyzed in depth in order to reveal methodological similarities and differences. Specifically, the studies were compared in terms of study objectives, setup and data acquisition, participants and experimental protocol, estimated gait parameters, statistical analysis, findings, and data availability.

### 3.1. Study Objectives

A first source of inhomogeneity concerned the primary objectives of the reviewed studies, which implicitly affected all the other aspects as well. For instance, in Latorre et al. [28], the main goal was to characterize gait by comparing five different methodologies to estimate gait parameters and using video analysis for validation purposes. In Latorre et al. [18], on the other hand, the primary purpose was to estimate gait parameters to check their correlation with some clinical tests commonly used on post-stroke subjects. The studies performed by Vernon et al. [25] and Clark et al. [87] were based on the same approach, whilst Ferraris et al. [5] had the primary goal of validating the proposed solution for gait characterization against a traditional gait analysis system. Finally, unlike the previous studies, Luo et al. [26] and Gao et al. [9] focused on characterizing walking patterns through objective parameters for classification purposes, using supervised classifiers and machine learning techniques, and on estimating a summary index of gait quality from joint trajectories, respectively. The differences in main objectives also led these studies to differ in methodology, experimental protocols, data analysis, and final outcomes.

### 3.2. Setup and Data Acquisition

As no studies using the latest generation device, the Azure Kinect, had been performed on hemiplegic patients so far, almost all of the selected studies used the Microsoft Kinect v.2 (second-generation model), with the exception of the studies by Clark et al. [87] and Vernon et al. [25], which were based on the Microsoft Xbox 360 (first-generation model) instead. All researches adopted a frontal view for motion capture during walking, since the frontal view of the walkway, and thus of the subject, was the one that optimized the accuracy of the body tracking algorithm [96,97] provided by the optical device and on which the motion analysis was based. The only exception was in Vernon et al. [25], where the camera position was slightly off-center with respect to the walkway. In contrast, Gao et al. [9] did not specify details regarding the camera position in the experimental setup. In Latorre et al. [28], the setup also included a second optical sensor (a simple RGB camera) positioned laterally and externally with respect to the walkway to obtain also a side view of the subject’s gait. The second camera was used solely for validation purposes through a video analysis method.

All studies adopted a straight walkway approximately 6 m long for motion capture and data acquisition. Given the operational requirements and field of view of the Kinect sensor, the area for the estimation of gait parameters is typically limited to a maximum of 4 m from the camera, with a minimum distance of about 0.5 m to allow for full-body acquisition. In all studies, data were acquired during a one-way walk toward the camera, as is done in traditional gait analysis. The only exception was Vernon et al. [25], where the TUG test was used instead, which involved several phases including getting up from the chair, walking a shorter round-trip distance (about 3 m), and sitting back down in the chair.

In terms of validation, only Ferraris et al. [5] adopted a traditional validation method, namely the comparison with a gold standard system. In Latorre et al. [28], an alternative validation method was adopted by exploiting the lateral RGB camera, a series of vinyl lines printed along the walkway, and a video labeling procedure: with this approach, it was possible to compare the estimated measurements with the real ones. The other studies reported no information regarding a validation procedure.

The last aspect of the setup concerned clothing. Some studies (Latorre et al. [28], Latorre et al. [18] and Vernon et al. [25]) reported constraints on clothing to optimize the performance of the body tracking algorithm, particularly the use of tight-fitting, light-colored, and non-reflective clothing. In Clark et al. [87], shoes and usual clothing were suggested, using a tape to make the pants tighter, if necessary, in order to make the joints of the skeletal model more stable. In Ferraris et al. [5], however, because of the need to apply retroreflective markers at reference points on the body for validation purposes, only underwear was worn. The other studies did not report information on clothing.

### 3.3. Participants and Experimental Protocol

Based on their specific objectives, the studies included enrolling participants according to well-defined inclusion criteria. Most studies included also a control group consisting of healthy subjects, with the exception of Ferraris et al. [5], Clark et al. [87], and Vernon et al. [25], in which only post-stroke subjects were considered. In the first study (Ferraris et al. [5]), the goal was to validate the proposed solution against a gold standard system; in the last two studies (Clark et al. [87] and Vernon et al. [25]), the goal was to investigate the correlation between gait parameters and specific clinical tests commonly used on post-stroke subjects. In some papers, control subjects were not included as the studies focused exclusively on a post-stroke population; this element could represent a limitation, as the presence of controls may support data for comparative purposes.

In Latorre et al. [28] and Latorre et al. [18], the control group included both young and elderly subjects. In the first study (Latorre et al. [28]), the objective was to compare five different methodologies for assessing spatiotemporal gait parameters. The second study (Latorre et al. [18]) aimed to evaluate the effect of age (divided into decades) on spatiotemporal parameters. Therefore, the inclusion of younger subjects in the control group was in line with the specific objectives of both studies.

In Luo et al. [26], healthy subjects were divided into two distinct groups (young and old subjects): this division was rather irrelevant considering that the ultimate goal of the study was to classify participants with and without hemiplegia through machine learning methods and supervised classifiers.

In Gao et al. [9], the control group matched the post-stroke subjects in age, as the purpose of the study was to compare the walking characteristics of the two groups.

Regarding the experimental protocol, all studies required participants to perform multiple walking trials to ensure repeatability. In Latorre et al. [28], Latorre et al. [18], and Ferraris et al. [5], the experimental protocol planned at least three repetitions; while nine repetitions were planned in Luo et al. [26]. Contrarily, in Gao et al. [9], no multiple walking trials were planned, but rather a continuous walk of at least 30 s. Additionally, in Vernon et al. [25] and Clark et al. [87], no number of walking repetitions was specified, but only two test sessions were separated by one week. Finally, no trials at different walking speeds were included in the selected studies: instead, all experimental protocols instructed participants to use a comfortable and normal walking velocity.

### 3.4. Estimated Gait Parameters

Gait assessment is commonly based on spatiotemporal parameters, as in traditional gait analysis with gold-standard systems. However, there is a certain level of inhomogeneity among studies in this regard. In particular, the spatiotemporal parameters considered are generally not the same. In addition, the method used to estimate gait parameters varies from study to study: this introduces a bias in the results that makes direct comparisons between studies complicated.

For example, in Latorre et al. [28] and Latorre et al. [18], some of the most commonly used spatiotemporal parameters in traditional gait analysis were estimated: gait speed, step information (distance and time), stride information (distance and time), asymmetry, double support, and swing time. In Latorre et al. [18], in addition to spatiotemporal parameters, some kinematic parameters were estimated and analyzed.

In contrast, Clark et al. [87] and Ferraris et al. [5] estimated only a subset of spatiotemporal parameters related to the body’s single side (right and left sides) and the overall walk. In addition, Ferraris et al. [5] included parameters related to the body’s center of mass, which could be relevant for identifying specific abnormalities during walking associated with increased risk of fall (e.g., walking patterns with relevant lateral body sways). For the same reason, Luo et al. [26] also used parameters related to the body center of mass, derived with a methodology similar to Ferraris et al. [5], as they could be significant for classifying subjects with and without hemiplegia.

Vernon et al. [25], on the other hand, estimated a small number of spatiotemporal parameters related only to the first step, the first stride, and the walking speed, as the setup used limited the space available for the gait analysis. Along a different line was Gao et al. [9], where no spatiotemporal parameters were estimated. Instead, a gait index was estimated from the analysis and overall motion (i.e., 3D trajectories) of specific joints in the skeletal model.

### 3.5. Statistical Analysis Methods

The statistical analysis is also closely linked to the objectives of a study, and for this reason, it generally includes several statistical tests that can, consequently, make the comparison of results more or less complex.

For example, in studies involving a validation procedure, the goal is to demonstrate the accuracy of the obtained measures compared to a gold-standard system. In fact, in Latorre et al. [28], the average values (with standard deviation) of the estimated parameters for the control group and the post-stroke subjects were reported, as well as the estimated mean square error compared to the video analysis, that is, the gold-standard for this study.

In Ferraris et al. [5], a series of statistical (Wilcoxon test) and correlation (Spearman correlation and ICC) tests were considered to demonstrate the accuracy of the estimated parameters on post-stroke subjects: estimated parameters were reported as median values with first quartile, compared to an optoelectronic system, the gold-standard for this study. In addition, Spearman’s correlation was used to investigate the correlation between the estimated parameters and the TUG test, administered before the walking trials.

Other studies mainly focused on the correlation between walking parameters and tests commonly used in clinical practice on post-stroke subjects, consequently using a statistical analysis that was more or less complex but appropriate to the study’s objectives. For example, because the study had multiple objectives, many statistical and correlation tests were used in Latorre et al. [18]: paired t-test to evaluate the significance of the statistical difference between populations divided by decade (control group and post-stroke subjects) and to identify subjects at risk of falling compared to the Berg balance scale; Pearson’s correlation coefficient to evaluate the effect of age on the estimated parameters and to validate the proposed solution against a set of clinical tests; and two-way random effect ICC to evaluate the inter-rater reliability between two raters and the intra-rater reliability for each individual rater.

In contrast, Vernon et al. [25] included only two statistical tests: ICC for the test-retest reliability between the estimated parameters and the clinical tests performed on post-stroke subjects in the two planned sessions; and Spearman’s correlation to evaluate the correlation between the estimated parameters and the TUG test. The same statistical tests were also included in Clark et al. [87], where the Spearman’s correlation was used to evaluate the correlation of estimated gait parameters with static and dynamic balance. In contrast, the studies by Luo et al. [26] and Gao et al. [9], which focused on classification, did not report information on statistical analysis.

### 3.6. Findings and Data Availability

As discussed in the previous points, the inhomogeneity of the study objectives led to different methodological approaches and thus to different results and findings.

Latorre et al. [28] characterized gait by comparing five different video analysis methodologies to estimate spatiotemporal parameters during gait, using video analysis for validation purposes. Following this approach, the overall results revealed limited accuracy between the Kinect-based and the video-based measurements in both the healthy and post-stroke groups. The authors hypothesized that such inaccuracies may be due to the speed and jitter of the tracking of ankle and toe [98,99], thus explaining the poor results achieved on the estimation of short duration and length event (e.g., double support) and the event that required the toe-off detection (e.g., swing time). Additionally, even though the use of video analysis is acknowledged as a valid approach in clinical setting, it may introduce additional errors in the measurements with respect to laboratory-grade systems [100]. According to the overall results, they concluded that the Kinect v2 could be used as a complementary tool to support the gait analysis in the estimation of events with a certain duration and length.

With regard to the validation of the optical device with respect to the gold-standard optoelectronic systems, the results achieved by Ferraris et al. [5] show good agreement, accuracy, and correlation between the subset of spatiotemporal parameters estimated by the two systems, and compared with a clinical assessment test (i.e., Timed Up and Go Test). The results suggest the reliability of an optical-based system for the evaluation of gait impairments, even though some aspects need to be further explored. For instance, the restricted number of participants may have biased the robustness of the characterization of the gait parameters, thus affecting the clinical and statistical findings.

Regarding the correlation of the Kinect-derived gait parameters with clinical tests for post-stroke patient assessment, the study presented by Latorre et al. [18] showed excellent intra-reliability between the clinical test and almost all gait measures, also allowing for the identification of patterns exposing the patient to fall risk. However, the minimal detectable change was inconstant among the measured gait parameters, resulting in a poor estimation of the kinematic parameters.

Similar results on the estimation of gait parameters were achieved by Vernon et al. [25] and Clark et al. [87]. In particular, in Vernon et al. [25], all Kinect-estimated variables showed excellent reliability (ICC > 0.90), with the exception of the trunk flexion angle. Similarly, as the two authors worked on the same dataset, the estimated variables confirmed the previously found high reliability (ICC > 0.80), even though many of the results were redundant.

The remaining studies focused on gait pattern characterization and classification through objective parameters using machine learning techniques. In particular, Luo et al. [26] worked on the development of a random forest method for the classification and analysis of hemiplegic gait. The method was developed starting from a pool of gait features (e.g., stride length, gait speed, left/right moving distances) acquired via Kinect, resulting in the achievement of an averaged classification accuracy of 90.65% among all the combinations of gait features.

Conversely, whereas Luo et al. [26] highlighted the usability of Kinect-derived data in machine learning techniques, Gao et al. [9] used the Kinect to record kinematic data during walking to obtain a quantitative evaluation of the gait quality of the hemiplegia group according to a gait quality index (GQI) based on a radar map. The final results show a significant negative correlation between the GQI and the Fugl-Meyer Assessment score for lower limbs, together with a significant statistical difference in lower limb joint movement quality between the healthy and the hemiplegia groups, reflecting the differences in motion quality of the joints for the two groups. The results thus highlight the reliability of the GQI, estimated from joint trajectories, as an assessment tool to support clinical decisions on rehabilitation programs.

## 4. Conclusions

The aim of this review was to give an overview of the state of the art in the use of a low-cost and non-invasive optical body tracking system, based on Microsoft Kinect, to assess gait in post-stroke individuals through an extensive search of the available literature. As a narrative review, it was aimed at summarizing the current state of the art on the presented application, in order to open a general debate on the topic and to underline the validity of such approaches, together with the current lack of knowledge. For this reason, it was chosen not to apply any standards for the critical appraisal of the studies’ quality [101], as this approach is typical of systematic reviews. In the future, to complete this work, it will be possible to write a systematic review using quantitative methods to assess the quality of the chosen works.

As previously discussed, the main limitation of the papers included in this review was related to a lack of homogeneity among the different studies in terms of methodology (experimental setup, parameters identification, statistical analysis) that prevented a full and accurate comparison of the results from several aspects, above all those regarding quantitative comparisons. At present, this fact, in our opinion, precludes the possibility of using clinical-statistical techniques for a metanalysis. In particular, differences in the primary goal led to significant differences in methodology, experimental setup, protocols, data analysis, and final findings. For instance, although most of the studies focused on investigating the correlation of gait parameters with specific clinical tests commonly used on post-stroke patients, they generally considered different spatiotemporal parameters estimated with different approaches and algorithms. Additionally, it should be noted that just one study [5] reported a traditional validation methodology versus a gold-standard system (i.e., optoelectronic system). Whilst the results revealed good agreement, accuracy, and correlations between the spatiotemporal gait parameters estimated by the two systems, it should be noted that Kinect-based estimation may sometimes have had limited accuracy and limited sensitivity to kinematic parameters [18].

Despite the lack of homogeneity among the different studies, independently from the different choice of parameters and estimation methods, our overview of selected studies showed the growing potential of using the Kinect sensor in human motion analysis, including the quantitative assessment of gait parameters, in patients with stroke. A limited number of studies used Kinect technology in these specific neurological patients to quantify the functional impairment during gait or the effects of rehabilitative programs. The use of Kinect for the rehabilitation of stroke patients is in fact a recent topic.

Although the Kinect-only based approach for motion analysis is not yet fully used to evaluate gait patterns in clinical settings, its use as a complementary tool with laboratory-grade systems is encouraged, as the obtained results demonstrated the usefulness of a Kinect-based gait analysis as a low-cost tool that can overcome the typical limitations of measurements in indoor laboratory environments, such as high cost, dependency on trained personnel, and the need to wear limited clothing.

It is important to highlight that researchers are very interested in devices such as the Kinect that were not originally designed for the purpose of medical research on walking assessment. This device provides in fact unparalleled access to a low-cost, markerless, non-invasive, portable, and easy-to-use solution for assessing the kinematic and spatiotemporal aspects of physical function in healthy and clinical populations. While studies have assessed the validity of the Kinect for over-ground gait assessment, in pathological populations such as Parkinson’s disease [77,88,102] and stroke [87], few have examined whether the data it provides could be beneficial in a clinical setting. Some studies have demonstrated the ability of Kinect to discriminate between healthy and pathological individuals [103]. However, its advantages should be weighed against its sensitivity, which, as reported by Latorre et al. [18], may be limited with respect to kinematic parameters, as well as in estimating more challenging spatiotemporal parameters, such as step width [5] or gait cycle phase [104]. Thus, although the full strengths and weaknesses of Kinect-based analysis methods need to be further investigated, the Kinect seems to be particularly suitable to be used also for post-stroke subjects as a non-invasive and easy-to-use motion analysis tool in environments where gold standard systems cannot be used, including ambulatory settings and private home environments, thus opening new perspectives for remote monitoring and rehabilitation strategies, especially in unsupervised settings. Rehabilitation, for instance, is less demanding regarding accuracy requirements. Video-based systems could provide adequate accuracy and a finer evaluation of gait patterns compared to clinical judgments, detecting changes not yet recognized by clinicians, scales or questionnaires. Clinicians may continue to use the clinical scales and tests that are familiar to them (such as the TUG test), but can also rely on an automated assessment of gait, consistent with the scales, which is able to provide significantly more information and allow detection of more changes. These aspects are particularly important for promptly modulating and personalizing the home rehabilitation program, for the reduction of cost effectiveness, thereby limiting the need for hospital evaluations and improving the patient’s quality of life at the same time.

## Figures and Tables

**Table 1:** Summary of the main details of the reviewed studies.

Source	Year and Country	# Participants, Age (yrs) and Gender (# M/F)	Height (cm) and Weight (kg)	Functional Tests	Gait Parameters	Finality of the Study
Vernon et al. [25]	2015 Australia	Total: 30 post-stroke 68 ± 15 yrs M: 21/F: 9	Height: 166.7 ± 9.4 Weight: 72.5 ± 11.9	Gait analysis (10 m walk) TUG (Timed Up and Go) FR (Functional Reach) ST (Step test)	Trunk flexion (deg) Flexion angle velocity (deg/s) Step length (m) Stride length (m) Gait speed (m/s) Turning time (s) Total time (s)	Characterization
Clark et al. [87]	2015 Australia	Total: 30 post-stroke 68 ± 15 yrs M: 21/F: 9	Height: 166.7 ± 9.4 Weight: 72.5 ± 11.9	Gait analysis (10 m walk) TUG (Timed Up and Go) FR (Functional Reach) ST (Step test)	Affected step length (mm) Unaffected step length (mm) Step length asymmetry (%) Affected foot swing velocity (m/s) Unaffected foot swing velocity (m/s) Foot swing velocity asymmetry (%) Mean velocity (m/s) Peak velocity (m/s) Peak–Mean velocity difference (%)	Characterization
Luo et al. [26]	2020 China	Total: 60 Hemiplegia patients: 20 54.3 ± 12. yrs M: 12/F: 8 Control group (healthy old): 20 71.83 ± 10.55 yrs M: 10/F: 10 Control group (healthy young): 20 24.43 ± 3.83 yrs M: 13/F: 7	Height: 164.75 ± 6.13 Weight: 61.5 ± 10.1 Height: 159.83 ± 10.49 Weight: 58.16 ± 7.52 Height: 169 ± 6.87 Weight: 59.93 ± 13.58	Gait Analysis (4 m walk test)	Stride length (m) Gait speed (m/s) L/R distance (m) Up/Down distance (m)	Characterization
Latorre et al. [28]	2018 Spain	Total: 83 Hemiplegia patients: 38 56.1 ± 13.2 yrs M: 22/F: 16 Control group: 45 30.6 ± 7.6 yrs M: 31/F: 14	Not reported	Gait Analysis (6 m walk test)	Gait speed (m/s) Stride length (m) Stride time (s) Step length (m) Step time (s) Step asymmetry (m) Double support time (s) Swing time (s)	Characterization
Latorre et al. [18]	2019 Spain	Total: 464 Hemiplegia patients: 82 48.3 ± 16.14 yrs M: 55/F: 27 Control group: 382 43.3 ± 18.6 yrs M: 169/F: 186	Not reported	BBS (Berg Balance Scale) DGI (Dynamic Gait Index) 1mWT (1-min walking test) Gait Analysis (10 m walk test)	Gait speed (m/s) Stride length (m) Stride time (s) Step length (m) Step time (s) Step width (m) Cadence (step/min) Step asymmetry (m) Double support time (s) Swing time (s) Angles (trunk, pelvis, hip, knee and ankle joints)	Characterization
Gao et al. [9]	2021 China	Total: 20 Hemiplegia patients: 15 41–60 yrs (average 49) M: 8/F: 7 Control Group: 15 42–62 yrs (average 48) M: 8/F: 7	Weight: 68.25 (range: 61–74) Height: 168.96 (range: 1.63–1.75) Weight: 69.82 (range: 62–76), Height: 169 (range: 164–176).	30 sWT (30 s walking test)	GQI (Gait Quality Index)	Characterization
Ferraris et al. [5]	2021 Italy	Hemiplegia patients: 11 53.3 ± 13.9 yrs M: 8/F: 3	Not reported	TUG (Timed Up and Go) Gait analysis	Step length (m) Stance duration (%) Double support duration (s) Mean velocity (m/s) Cadence (step/min) Step width (m)	Validation and Characterization
